# Overexpression of the lncRNA HOTAIRM1 promotes lenvatinib resistance by downregulating miR-34a and activating autophagy in hepatocellular carcinoma

**DOI:** 10.1007/s12672-023-00673-8

**Published:** 2023-05-12

**Authors:** Danyan Gu, Meng Tong, Jing Wang, Bocheng Zhang, Jinghua Liu, Guoqiang Song, Biao Zhu

**Affiliations:** 1 Department of Critical Care, Fudan University Shanghai Cancer Center, Shanghai, 200032 China; 2 Department of Oncology, Shanghai Medical College, Fudan University, Shanghai, 200032 China; 3 Department of General Surgery, Jinzhou Medical University, Jinzhou, 121001 China; 4 Department of Radiology, Linyi People’s Hospital, Linyi, 276000 China; 5 Department of Hepatobiliary Surgery and Minimally Invasive Institute of Digestive Surgery and Prof. Cai’s Laboratory, Linyi People’s Hospital, Linyi, 276000 China; 6 Department of Pulmonary, Department of Cancer Center, Changxing Hospital of Traditional Chinese Medicine, Huzhou, 313100 China

**Keywords:** Hepatocellular carcinoma (HCC), Long noncoding RNAs (lncRNAs), HOTAIRM1, Lenvatinib, miR-34a

## Abstract

**Background:**

Hepatocellular carcinoma (HCC) is one of the most common malignant cancers in humans and has a high fatality rate. Despite pharmacological advances such as sorafenib and lenvatinib approval, responses are seen only in a limited fraction of HCCs, and the majority of HCC patients do not benefit from this treatment. In recent years, researchers have verified that the long noncoding RNAs (lncRNAs) impact the efficiency of lenvatinib and the prognosis of patients with HCC.

**Materials and methods:**

This work obtained gene expression profile from an Arraystar lncRNA microarray. Expression of HOTAIRM1, Beclin-1, and p62 in HCC was characterized in clinical HCC tissues of 24 patients with HCC. Overexpression and knockdown experiments were performed in HCC cells to examine the effects of the HOTAIRM1 on lenvatinib sensitivity. The interactions between HOTAIRM1, miR-34a and Beclin-1 were predicted according to GSEA and CNC network. The effects of HOTAIRM1, autophagy and lenvatinib on tumor inhibit were validated in orthotopic tumor-bearing nude mouse model.

**Results:**

Lenvatinib-resistant HCC cell lines were established using the concentration gradient method. Data from an Arraystar lncRNA microarray indicated that HOTAIRM1, a specific lncRNA located in an evolutionarily highly conserved HOX gene cluster, was differentially expressed between lenvatinib-resistant HCC cells and their parental cells. Expression of HOTAIRM1 and Beclin-1 in HCC was characterized in clinical HCC tissues of 24 patients who have different sensitivity to lenvatinib. Knocking down of HOTAIRM1 decreased the autophagy level in lenvatinib-resistant HCC cells and increased their sensitivity to lenvatinib, especially when combined with autophagy inhibitors both in vitro and in vivo. Further study indicated that knocking down HOTAIRM1 in lenvatinib-resistant cell lines increased the level of miR-34a and inhibited the expression of Beclin-1 in Huh7-R and HepG2-R cells. Investigation according to GSEA and CNC network, lncRNA and nearby coding gene and lncRNA-miRNA analyses demonstrated that the resistance of HCC to lenvatinib was affected by the HOTAIRM1-miR-34a-Beclin-1 regulatory axis.

**Conclusion:**

HOTAIRM1 is an independent drug resistance factor which significantly associated with the efficacy of lenvatinib in HCC. HOTAIRM1 may downregulation of miR-34a and upregulation of Beclin-1, leading to activation of autophagy, thereby inducing lenvatinib resistance in HCC.

**Supplementary Information:**

The online version contains supplementary material available at 10.1007/s12672-023-00673-8.

## Introduction

Hepatocellular carcinoma (HCC) is one of the most common tumors and one of the leading causes of cancer death in the world [1]. With an increasing global incidence, the latest statistics show that liver cancer is the third leading malignant tumor and the second leading cause of cancer death in China [2]. Due to the insidious onset of HCC and the difficulty of early diagnosis, most patients have already reached an advanced stage or even developed distant metastasis at the time of diagnosis. The surgical resection rate is low, and the prognosis is poor [3]. With the rapid development of modern tumor biotherapy, molecularly targeted drugs have attracted increasing attention and have gradually demonstrated their unique antitumor effects [4]. Sorafenib is the first molecularly targeted drug approved by the Food and Drug Administration (FDA) for the treatment of advanced HCC [5]. Existing clinical evidence shows that sorafenib has significantly improved the prognosis of HCC patients in the past decade [6]. However, many HCCs respond poorly to sorafenib or develop resistance after months of treatment. In 2018, lenvatinib showed an increased survival in HCC patients and became the first therapeutic alternative to sorafenib [7]. However, the emergence of drug resistance severely limits its clinical application, but the mechanisms are complicated [8–10].

Long noncoding RNAs (lncRNAs) have been reported to participate in multiple cellular biological processes, such as the cell cycle, cell differentiation, cell apoptosis, and autophagy. lncRNAs are also one of the most popular topics in the study of drug resistance in HCC [11]. The lncRNA HOTAIRM1, located between the HOXA1 and HOXA2 genes, is best known for its function in activating the HOXA genes in neural differentiation of pluripotent cells [12, 13] and differentiation professes of promyelocytic leukemia cells [14]. However, altered HOTAIRM1 expression has now been reported in lots of human cancers [15]. But the mechanism of HOTAIRM1 in cancer is largely unknown. Recent evidence has shown that HOTAIRM1 plays an important role in treatment resistance in different cancers. For example, Ahmadov et al. reported that HOTAIRM1 promotes tumor aggressiveness and radiotherapy resistance in glioblastoma [16]. Kim et al. proved that HOTAIRM1 promotes tamoxifen resistance by mediating HOXA1 expression in ER + breast cancer cells [17]. While in other study, HOTAIRM1 was thought playing a completely opposite role. Ren et al. first reported that HOTAIRM1 suppresses cell progression via sponging endogenous miR-17-5p in colorectal cancer cells [18]. However, there are no researches on the relationship between HOTAIRM1 and HCC drug resistance so far. Therefore, the identification and understanding of HOTAIRM1 will provide insights into HCC drug resistance for developing more effective therapeutic strategies.

In our study, we found that lncRNA HOTAIRM1 is critical for maintenance and spread of lenvatinib resistance in HCC. We demonstrated that HOTAIRM1 is upregulated in lenvatinib-resistant HCC cells and knockdown of HOTAIRM1 significantly increases lenvatinib sensitivity in HCC. Mechanistic studies further revealed that HOTAIRM1 downregulation of miR-34a and upregulation of Beclin-1, leading to activation of autophagy. Moreover, we discovered that HOTAIRM1 knockdown and a combinatorial strategy using autophagy inhibitor therapy may reprogram the tumor microenvironment for effective treatment of lenvatinib-resistant HCC.

## Materials and methods

### Clinical specimen collection

In this study, 24 patients (aged 41–55 years, an average age of 47.21 ± 6.15 years) who were pathologically and clinically diagnosed as HCC in Linyi People’s Hospital from December 2018 to December 2022, including 18 males and 6 females, were randomly selected. Consent forms were obtained from all patients, and the experimental protocol was approved by the local ethics committee. Abdominal US, computed tomography and magnetic resonance imaging were used to evaluate the tumors. The patient was classified on the basis of tumor size and TNM stage in line with American Joint Committee on Cancer tumor staging system (seventh edition). Among patients, 17 positive for the hepatitis B surface antigen and 1 positive for the HCV, other 6 were negative for both. Most of the HCCs were associated with high alpha-fetoprotein (AFP) (19 patients in all 24). All of the enrolled patients were received only lenvatinib for treatment. The patients who received other therapies, such as chemotherapy, radiotherapy, or immune therapy, and who were not diagnosed by pathology were excluded. The cancerous tissues were collected from the enrolled patients with HCC when taking the biopsy. Tissue samples were frozen in liquid nitrogen and cryopreserved at − 80 °C for subsequent experiments. The expression levels of HOTAIRM1, Beclin-1 and p62 in different lenvatinib-sensitive HCC samples were detected by real-time quantitative PCR (RT‒qPCR). The complete follow-up data till disease progression after treatment were collected. Multivariate analyses of factors showed that no significant association between HOTAIRM1 expression and poor differentiation grade, liver cirrhosis, advanced TMN stage, tumor size, AFP, Child score and etiologies (HBV and HCV).

### Cell line resources

Two human HCC cell lines (Huh7 and HepG2) were purchased from the Cell Bank of the Type Culture Collection of the Chinese Academy of Sciences, Shanghai Institute of Cell Biology, Chinese Academy of Sciences.

### Cell culture

Two human HCC cell lines (Huh7 and HepG2) cultured according to the supplier's instructions. Huh7 and HepG2 cells were cultured in DMEM medium with 10% fetal bovine serum (FBS) at 37 °C and 5% CO2 (DMEM: Gibco Invitrogen, Carlsbad, CA, USA).

### Establishment of lenvatinib-resistant HCC cell lines (named Huh7-R and HepG2-R) using the concentration gradient method

To examine possible lncRNAs involved in mediating lenvatinib resistance in HCC, we first established two lenvatinib-resistant HCC cell lines (Huh7-R and HepG2-R) by culturing cells with escalating doses of lenvatinib (0.1–3 μM for Huh7-R cells and 0.1–5 μM for HepG2-R cells) for 1 year. Cells in the logarithmic growth phase were added to the medium with a lower initial concentration of lenvatinib. After culturing for 24 h, the medium was discarded, washed, and replaced with fresh medium without lenvatinib. After the cells recovered, they were digested and passaged again. The cells were treated with low concentrations of lenvatinib for another 24 h. After the cells grew stably at this concentration, the concentration of lenvatinib was gradually increased to continue culture, and the above steps were repeated to establish lenvatinib-resistant HCC. The established Huh7-R and HepG2-R cells were verified by cell viability assay (MTT) as described below. Absorbance at 450 nm was measured using a microplate reader (Bio-Rad, Hercules, CA, USA). We then confirmed lenvatinib resistance in these two cell lines by comparison with their parental cell lines. The data indicated that the two lenvatinib-resistant cell types were insensitive to lenvatinib and exhibited a faster growth rate under lenvatinib treatment than their parental cells.

### Antibodies and inhibitors/agonists

Rabbit mAb to LC3I antibody (12741, dilution 1/800), rabbit mAb to p62 (23214, dilution 1/1000), rabbit anti-Beclin-1 (3495, diluted 1/1000), rabbit anti-Phospho-ULK1 (14202, diluted 1/800) and ULK1 (8054, diluted 1/1000) were all purchased from Cell Signaling Technology, Inc. Rabbit polyclonal anti-LC3II antibody (ab63817, dilution 1/1000) was purchased from Abcam. Anti-actin was obtained from Epitomics, an Abcam company (Cambridge, MA, USA). miR-34a inhibitors (5’-AGCCUUGCUGCAGGUGCGCAU-3’) and a nonsense control (NC) sequence (5’-UGCCUUACUGACGGUCGGAGA-3’) were obtained from Shanghai GeneChem, Inc. (Shanghai, China). According to the protocol, Huh7-R and HepG2-R cells (1 × 10^5^) were transfected with 50 nM miR-34a inhibitors, 50 nM miR-34a mimics, 50 nM scramble control inhibitors or 50 nM scramble control mimics using Lipofectamine® 2000 (Thermo Fisher Scientific, Inc.) for ~ 30 min at room temperature. After 48 h of transfection, the transfected cells were used for subsequent experiments. 3-Methyladenine (3-MA), a class III phosphoinositol 3-kinase (PI3K) inhibitor, and choroquine (CQ), were used as selective inhibitors of autophagy, was purchased from Santa Cruz Biotechnology (Texas, U.S.A). Rapamycin (rapa), a potent and specific mTOR inhibitor used as an autophagy agonist, was obtained from Selleck.

### Arraystar lncRNA microarray detection and bioinformatics analysis

Three duplicate groups of established lenvatinib-resistant HCC cells and their corresponding parental cells (Huh7 vs. Huh7-R and HepG2 vs. HepG2-R) were subjected to Arraystar lncRNA microarray analysis. Multiple lncRNAs and mRNAs that were differentially expressed in lenvatinib-resistant cells and their corresponding parental cells were screened. Bioinformatics methods of cluster, GO and pathway analyses were used to analyze the relationship between differential lncRNAs and mRNAs, combined with siRNA/shRNA technology, to identify the key molecules associated with lenvatinib resistance and the key molecules that regulated autophagy.

### RNA extraction and real-time qPCR

Total RNA was extracted from cultured cells and tumor tissues by using an ultrapure RNA extraction kit (CW Biotech, Beijing, China). cDNA was synthesized with an iScript cDNA Synthesis kit (Bio-Rad, CA, USA). Genomic DNA removal was performed at 42 °C for 2 min. The reverse transcription reaction was performed under the following conditions: 50 °C for 15 min and then 85 °C for 5 s. Real-time quantitative PCR was performed using the ABS-7500 Real-Time PCR System (Invitrogen Life Technologies, USA), and SYBR Green Mixed Low ROX (Bio-Rad) was used for product detection. The following cycling program conditions were used: 5 min at 95 °C, then 40 cycles of 15 s at 95 °C and 30 s at 60 °C. Primers used for the above experiments are shown in Table 1. All samples were run in triplicate in each experiment.

**Table 1:** The primers used for the RT-qPCR experiments

Gene	Primer	Sequence
HOTAIRM1	Forward Primer	*ATTTGGAGTGCTGGAGCGA*
	Reverse Primer	*CGCCAGTTCATCTTTCATTG*
ULK1	Forward Primer	*CCTTCCCATGCAGGCAACATATAAGC*
	Reverse Primer	*AGAAGCCGAAGGTTTCTTGGG*
p62	Forward Primer	*TGCTCTTCGGAAGTCAGCAA*
	Reverse Primer	*CCCGACTCCATCTGTTCCTC*
Beclin-1	Forward Primer	*AATCTAAGGAGTTGCCGTTATAC*
	Reverse Primer	*CCAGTGTCTTCAATCTTGCC*
miR-34a	Forward Primer	*ACACTCCAGCTGGGTGGCAGTGTCTTAGCT*
	Reverse Primer	*CTCAACTGGTGTCGTGGAGTCGGCAATTCAGTTGAGACAACCAG*
β-actin	Forward Primer	*CATGTACGTTGCTATCCAGGC*
	Reverse Primer	*CTCCTTAATGTCACGAT*

### Determination of cell viability

The MTT assay was used to measure cell viability. Negative controls and treated cells were seeded in 96-well flat bottom plates at a density of 200 cells/well. After culturing for 24 h, the original medium was replaced with fresh medium containing a certain concentration of lenvatinib and cultured at 37 °C and 5% CO2 for 2, 4 or 6 days, and MTT was added to produce crystal violet and was then discarded. Absorbance at 450 nm was measured using a microplate reader (Bio-Rad, Hercules, CA, USA).

### siRNA for Huh7-R and HepG2-R to knock down HOTAIRM1

Small interfering RNAs (siRNAs) were used to knock down HOTAIRM1 in Huh7-R and HepG2-R cells. The following siRNA and NC sequences were developed in a previous study [16]: HOTAIRM1 siRNA 1 sequence 5′-CCGTTCAATGAAAGATGAA-3′; HOTAIRM1 siRNA 2 sequence 5′-CCTGGAGACTGGTAGCTTA-3′; and NC sequence 5′-CCTAAGGTTAAGTCGCCCTCG-3′. Huh7-R and HepG2-R cells were seeded in 24-well plates and cultured in complete medium without streptomycin/penicillin. Cells were transiently transfected with 50 μmol/ml siRNA or an NC sequence with Lipofectamine 3000 (Cat#: L3000001, Thermo Fisher Scientific, USA). The inhibitory effect of lenvatinib on cell proliferation before and after knockdown of HOTAIRM1 was detected by MTT assay.

### Constructions of plasmid and cell transfection to knock down/overexpress HOTAIRM1

HOTAIRM1 overexpression plasmids, HOTAIRM1- knockdown plasmids (HOTAIRM1 shRNA with a corresponding negative control shRNA-NC) were designed by Genechem (Shanghai, China). According to the manufacturer’s protocol, the HCC cell lines Huh7-R and HepG2-R were infected with HOTAIRM1-knockdown shRNA or negative control shRNA by using Lipofectamine 2000 (Invitrogen, Carlsbad, CA, USA). The GV248 vector was used, and the stable clones were selected for further study by 5 μg/ml puromycin-containing medium. After 4 weeks selection, the puromycin-resistant cell clones were established. Gene-expression level was evaluated by RT‒qPCR.

### Western blotting

The levels of autophagy-related genes such as LC3II/I, p62, Beclin-1, p-ULK1 and ULK1 were detected by Western blotting. Transfected cells were collected, and the cell lysates were prepared and stored at − 80 °C until use. Protein concentration was determined using the BCA assay kit (BCA, Thermo Fisher, USA). After denaturation, the proteins in the samples were separated by gel electrophoresis using 8–12% SDS‒PAGE and transferred to PVDF membranes for approximately 1–2 h. The membrane blots were blocked with 5% skim milk. Blots were subsequently washed with TBS-T, incubated with the appropriate antibody (anti-rabbit/mouse, described above) overnight at 4 °C, washed three times with TBS-T and incubated with the indicated secondary antibodies (anti-rabbit/mouse, 7074/7056, CST) at room temperature for 2 h. The blots were rewashed with TBS-T and visualized using an ECL chemiluminescent substrate according to the manufacturer’s instructions (Pierce-Thermo Fisher Scientific, Waltham, MA, United States). Actin served as a positive control.

### Orthotopic tumor-bearing nude mouse model

An orthotopic model was developed for in vivo therapeutic efficacy studies. Approximately 1 million overexpressing HOTAIRM1 Huh7-R cells or shHOTAIRM1 Huh7-R cells (1:1) in Matrigel (Mediatech/Corning, Manassas, VA) were grafted into the left extrahepatic lobe of nude mice (6–8 weeks old). Tumor growth was monitored by high-frequency ultrasonography every 3 days according to the animal protocol. When tumors reached ~ 5 mm in diameter, mice were randomly assigned to a treatment group. The orthotopic model was then treated with lenvatinib monotherapy (30 mg/kg/day of lenvatinib administered orally for 15 consecutive days) and lenvatinib in combination with autophagy inhibitors (20 mg/kg/day of 3-MA intraperitoneal injection for consecutive 15 days)/agonists (1.5 mg/kg every 3 days of rapamycin intraperitoneal injection for consecutive 15 days).

Moribund status was used as the endpoint and the mice were then sacrificed if meet one of the following conditions, ≥ 10% weight loss compared with the starting date, or/and ≥ 10 mm in diameter of tumor size. tumor volume was calculated according to the formula below: tumor volume (mm3) = length (mm)*width (mm)* width (mm)/2. The animal study conformed to the principles of the Declaration of Helsinki and was approved by the Institutional Review Board of the Linyi People’s Hospital (Permit Number: LY2022058).

### Statistical analysis

Statistical analysis was performed using SPSS 21.0 (IBM, Armonk, NY, USA). Data were expressed as mean ± standard deviation (SD). Data between two groups were compared using student t test. Data among multiple groups were compared by one-way analysis of variance (ANOVA) with Tukey’s post hoc test. Data comparison between groups at different time points was performed using repeated-measures ANOVA with Bonferroni post hoc test. The correlation between HOTAIRM1 and clinicopathological characteristics of HCC patients was analyzed by chi-square test or Fisher’s exact test. The survival rate of mice was analyzed by Kaplan–Meier method. The significance level was defined as p < 0.05.

## Results

### Establishment of Huh7-R and HepG2-R cell lines

**Fig. 1:**
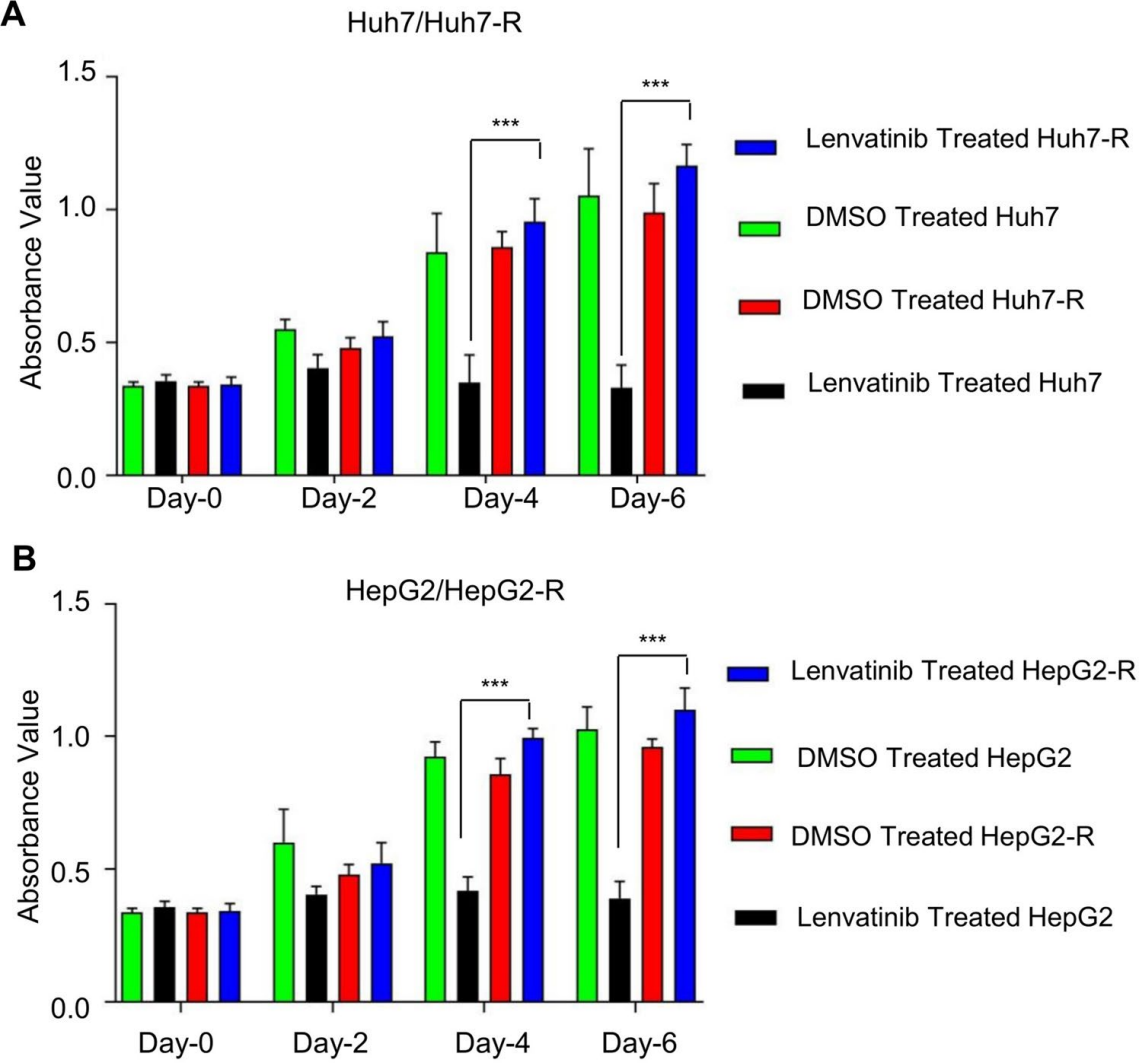
Proliferation inhibitory effect of lenvatinib on lenvatinib-resistant (HepG2-R and Huh7-R) and parental HCC cells (HepG2 and Huh7). Lenvatinib (1 μmol/L) significantly inhibited the proliferation of Huh-7 cells on days 0, 2, 4 and 6 (**A**), and 2 μmol/L lenvatinib significantly inhibited the proliferation of HepG2 cells on days 0, 2, 4 and 6 (**B**). Lenvatinib at the same concentration could not inhibit the proliferation of lenvatinib-resistant Huh7-R and HepG2-R cells. *** P < 0.001

To investigate the mechanism of lenvatinib resistance in HCC, lenvatinib-resistant HCC cell lines were established using the concentration gradient method. Two HCC cell lines, Huh7-R (resistant to 1 μmol/L lenvatinib) and HepG2-R (resistant to 2 μmol/L lenvatinib), were established, and cell viability was confirmed by MTT. With 1 μmol/L lenvatinib, the proliferation of Huh7 cells on days 0, 2, 4, 6 and 7 were significantly inhibited. The proliferation of lenvatinib-resistant Huh7-R cells under the same concentration of lenvatinib were not affected by lenvatinib (Fig. 1A and Additional file 1: Fig. S1A). With 2 μmol/L lenvatinib, the proliferation of HepG2 cells on days 0, 2, 4, 6 and 7 were significantly inhibited. The proliferation of lenvatinib-resistant HepG2-R cells under the same concentration of lenvatinib were not affected by lenvatinib (Fig. 1B and Additional file 1: Fig. S1B). The IC50 values of lenvatinib for different timelines of treatment are shown in Table 2. The results suggest that the established lenvatinib-resistant HCC cells are more resistant to lenvatinib than their parental cells.

**Table 2:** IC50 (μM) of drug-resistant and parental cell lines on Lenvatinib on different days

Time	Huh7	Huh7-R	HepG2	HepG2-R
Day2	1.699	3.898	2.109	5.762
Day4	0.989	2.781	1.263	4.123
Day6	0.205	1.942	0.301	2.783

### LncRNA HOTAIRM1 was highly expressed in lenvatinib-resistant cells

**Fig. 2:**
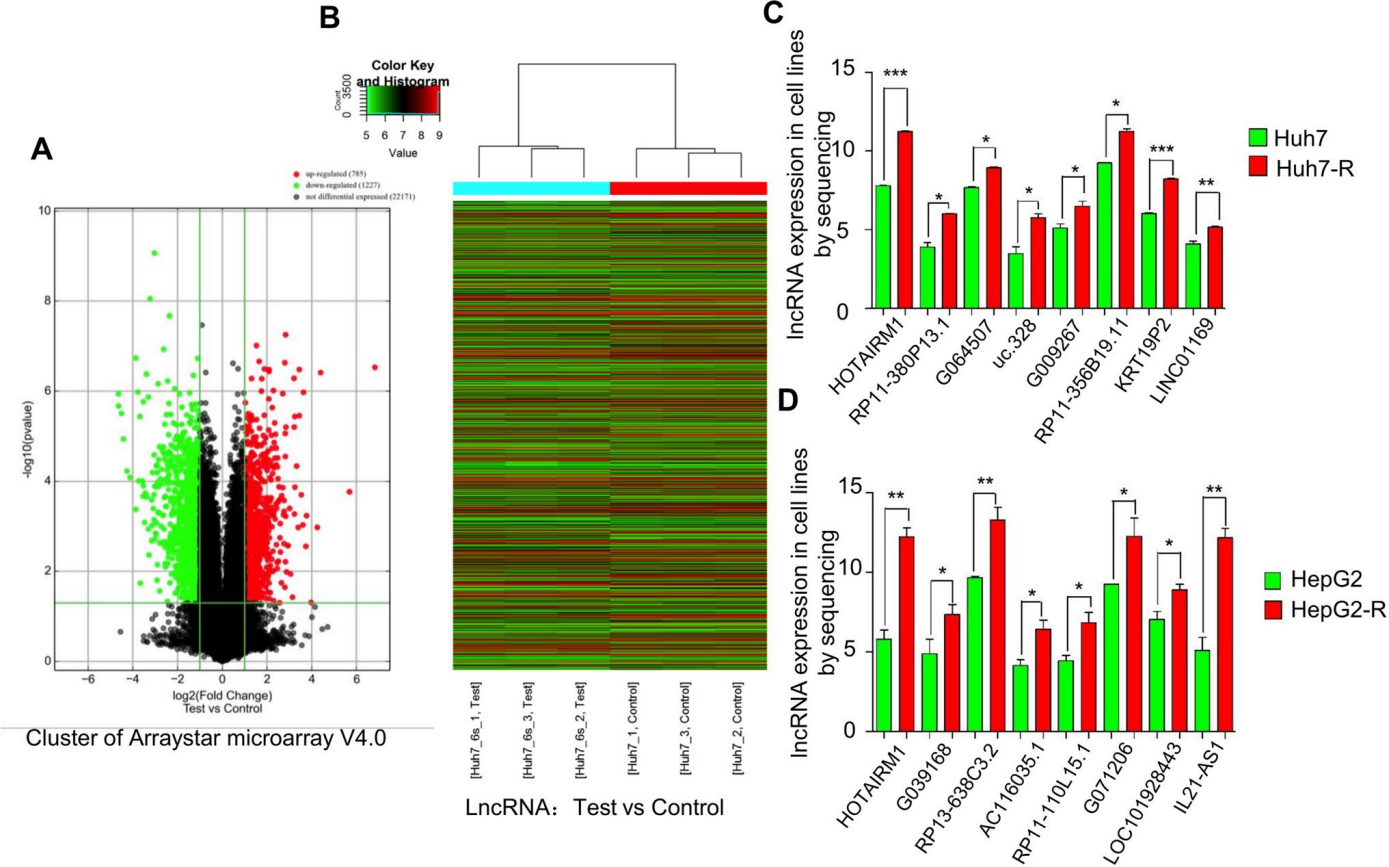
Differential expression profiles of lncRNAs in lenvatinib-resistant (HepG2-R and Huh7-R) and parental HCC cells (HepG2 and Huh7). Volcano plot of lncRNAs (**A**). The red dots indicate upregulated genes, and the green dots indicate downregulated genes. LncRNA cluster (**B**), Test vs. Control. More than 30,000 lncRNAs in total were detected with this chip. LncRNA HOTAIRM1 was significantly highly expressed in lenvatinib-resistant Huh7-R (**C**) and HepG2-R (**D**) cells. * P < 0.05, ** P < 0.01 and *** P < 0.001

To examine possible lncRNAs involved in mediating lenvatinib resistance in HCC, three duplicate groups of the established lenvatinib-resistant HCC cells and their corresponding parental cells (Huh7 vs. Huh7-R and HepG2 vs. HepG2-R) were subjected to Arraystar lncRNA microarray analysis. A volcano plot indicated many up- and downregulated genes in lenvatinib-resistant Huh7-R cells compared with the parental cells (Fig. 2A). Arraystar lncRNA microarray analysis detected more than 30,000 lncRNAs (Fig. 2B). Cluster analysis revealed that there were 1126 differentially expressed lncRNAs, accounting for approximately 3.7% of all lncRNAs. Among them, 797 were upregulated by ≥ twofold and 329 were downregulated by ≥ twofold. Bioinformatic analysis showed that the differentially expressed lncRNAs were related to gene transcription, autophagy, and other related regulatory processes. LncRNA HOTAIRM1 was one of the most significantly highly expressed genes in lenvatinib-resistant Huh7-R (Fig. 2C) and HepG2-R (Fig. 2D) cells compared with their parental cells. To validate the differentially expressed genes screened by the Arraystar lncRNA microarray, RT‒qPCR was used to verify the selected candidate lncRNAs in the lenvatinib-resistant cells and their parental cells. The results showed that HOTAIRM1 was also the most significantly highly expressed lncRNA in the lenvatinib-resistant Huh7-R (Additional file 2: Fig. S2A) and HepG2-R cells (Additional file 2: Fig. S2B), which was consistent with the microarray data, confirming that HOTAIRM1 may be a key lncRNA for lenvatinib resistance in HCC.

### Knockdown of HOTAIRM1 reverses lenvatinib-resistant HCC to lenvatinib-sensitive HCC via regulation of autophagy and vice versa.

**Fig. 3:**
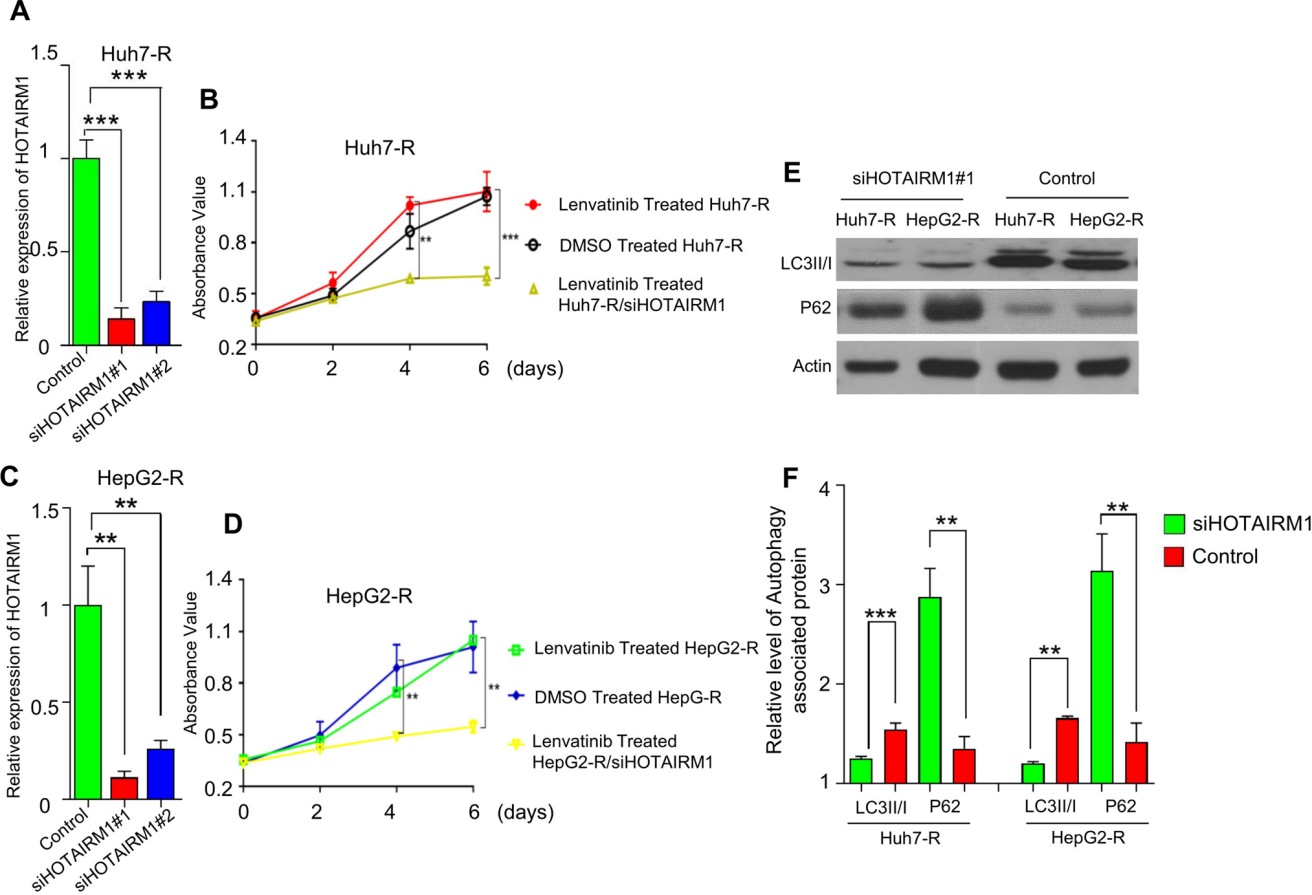
Knockdown of HOTAIRM1 reverses lenvatinib sensitivity in HepG2-R and Huh7-R cells by regulating autophagy. RT‒qPCR results showed that the expression level of HOTAIRM1 was significantly downregulated after siRNA transfection of lenvatinib-resistant cells compared with the control cells (**A**, **C**). MTT assay demonstrated the inhibitory effect of lenvatinib on cell proliferation with and without siRNA transfection of HOTAIRM1 (**B**, **D**). Western blotting showed that the level of the autophagy-related protein LC3II/I was significantly decreased after knockdown of HOTAIRM1, while the P62 level was significantly increased (**E**, **F**). ** P < 0.01; *** P < 0.001

To explore the function of HOTAIRM1 in lenvatinib-resistant HCC, siHOTAIRM1 and the NC sequence were transfected into lenvatinib-resistant cells (Huh7-R and HepG2-R). Forty-eight hours after transfection, HOTAIRM1 was significantly downregulated in Huh7-R (Fig. 3A) and HepG2-R (Fig. 3C) cells transfected with siHOTAIRM1 but not in cells transfected with the NC sequence. The inhibitory effect of lenvatinib on cell proliferation before and after knockdown of HOTAIRM1 was detected by MTT assay. The proliferation of Huh7-R cells transfected with siHOTAIRM1 under treatment with 1 μmol/l lenvatinib on days 0, 2, 4 and 6 were significantly inhibited. While the proliferation of Huh7-R cells transfected with the NC sequence under the same concentration of lenvatinib were not inhibited by Lenvatinib (Fig. 3B). The proliferation of HepG2-R cells transfected with siHOTAIRM1 under treatment with 2 μmol/l lenvatinib on days 0, 2, 4 and 6 were significantly inhibited. While the proliferation of HepG2-R cells transfected with the NC sequence under the same concentration of lenvatinib were not inhibited by Lenvatinib (Fig. 3D). All the above data indicated that knockdown of HOTAIRM1 reversed lenvatinib-resistant HCC to lenvatinib-sensitive HCC.

In recent years, researchers have verified that the autophagy signaling pathway impacts the efficiency of lenvatinib and the prognosis of patients with HCC [19]. Some studies have also shown that lncRNAs play an important role in autophagy [20]. The latest study even reported that HOTAIRM1 promoted autophagy and proliferation both in vitro and in vivo in leukemia cells [21]. In our current study, knockdown of HOTAIRM1 significantly reduced the expression of LC3II/I while increasing the protein level of p62 in both Huh7-R and HepG2-R cells (Fig. 3E, F). These data further suggest that HOTAIRM1 may trigger the autophagy pathway and lead to lenvatinib resistance in HCC.

To further verify if upregulate of HOTAIRM1 can reverses lenvatinib-sensitive HCC to lenvatinib-resistance HCC, parental sensitive cells HepG2 and Huh7 were examined by use of the autophagic flux inhibitors CQ before and after overexpression of HOTAIRM1. Overexpression of HOTAIRM1 reverses lenvatinib resistance in HepG2 and Huh7 cells by regulating autophagy. RT‒qPCR results showed that the expression level of HOTAIRM1 was significantly upregulated after transfection of HOTAIRM1 overexpression lentiviruses to lenvatinib-sensitive cells compared with the control cells (Additional file 3: Fig. S3A and C). MTT assay demonstrated the inhibitory effect of lenvatinib on cell proliferation with and without HOTAIRM1 overexpression lentiviruses transfection. And this effect can be reversed when autophagic flux inhibitors CQ was added (Additional file 3: Fig. S3B and D). *** P < 0.001.

### Beclin-1 was highly expressed in lenvatinib-resistant HCC and positively regulated by HOTAIRM1

**Fig. 4:**
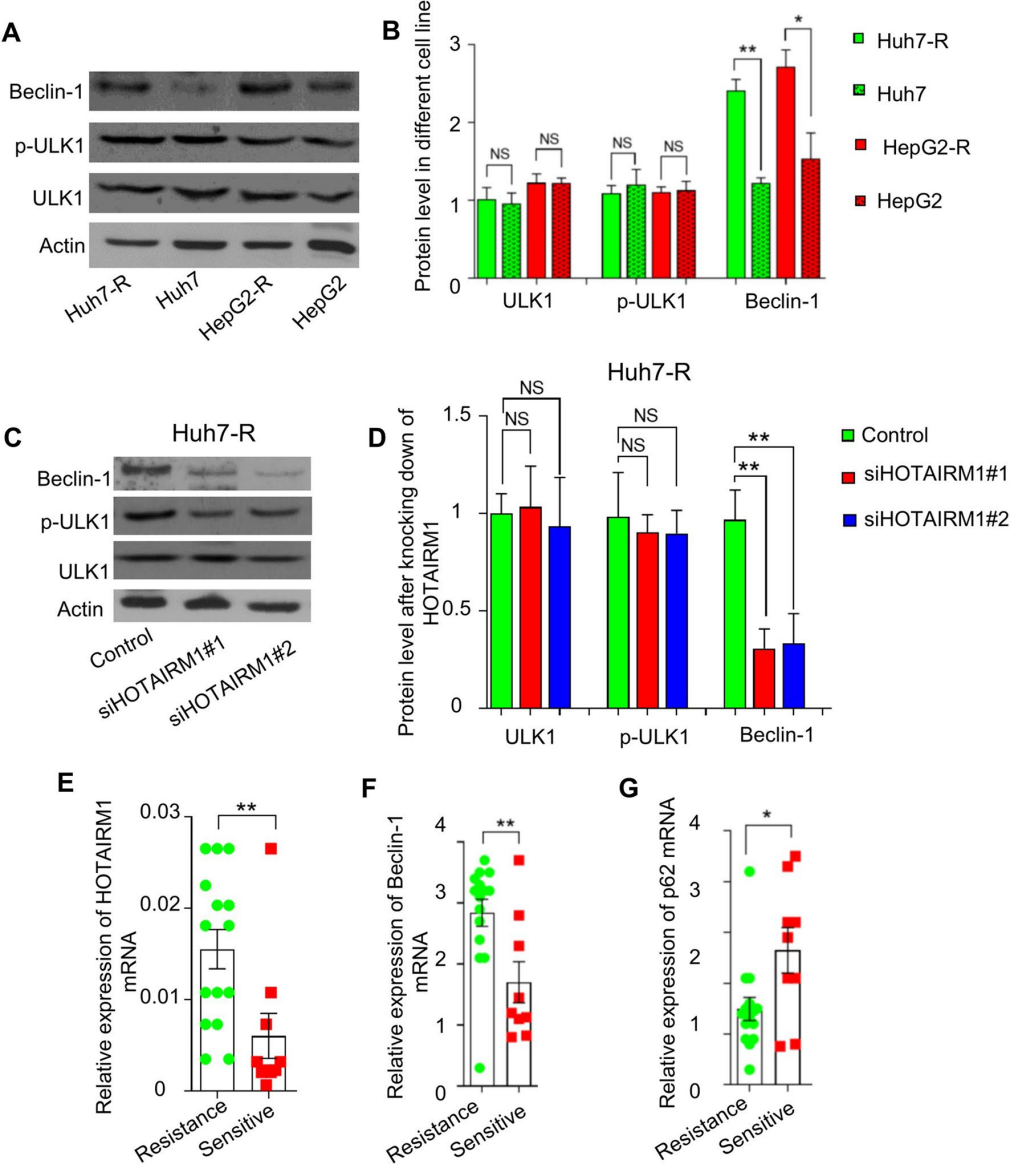
The levels of HOTAIRM1 and key autophagy-related molecules (ULK1, p-ULK1, and beclin-1) in HCC with different lenvatinib sensitivities. Western blotting showed that beclin-1 was significantly increased in Huh7-R and HepG2-R cells compared with their parental cells, but ULK1 and p-ULK1 were not (**A**, **B**). Knockdown of HOTAIRM1 significantly downregulated the mRNA level of beclin-1, while the mRNA expression of ULK1 and p-ULK1 did not change significantly (**C**, **D**). RT‒qPCR was performed to detect the mRNA levels of HOTAIRM1, beclin-1 and P62 in HCC tumor tissues from lenvatinib-resistant patients (N = 15) and lenvatinib-sensitive patients (N = 9). Lenvatinib-resistant patients demonstrated higher levels of HOTAIRM1 (**E**) and beclin-1 (**F**) and lower levels of P621 (**G**). * P < 0.05, ** P < 0.01, NS: no significant difference

To identify the key pathway that causes HCC lenvatinib resistance and the key molecules that mediate the regulation of autophagy by HOTAIRM1, bioinformatics methods were used to perform cluster analysis, GO and pathway analysis of the differential expression of lncRNAs and mRNAs. Some differentially expressed autophagy-related molecules, ULK1 and p-ULK1, and the autophagy-regulated marker molecule beclin-1 were identified. The Western blotting results showed that the expression levels of Beclin-1 in the lenvatinib-resistant Huh7-R and HepG2-R cells were significantly higher than those in the parental Huh7 and HepG2 cells (Fig. 4A and B). The protein levels of ULK1 and p-ULK1 were not significantly different between lenvatinib-resistant cells and their corresponding parental cells (Fig. 4A and B, Additional file 4: Fig. S4A). To further verify the potential relationship between the autophagy-related protein Beclin-1 and lncRNA HOTAIRM1, siRNAs were used to knockdown HOTAIRM1 in Huh7-R cells. Western blotting results showed that knockdown of HOTAIRM1 significantly reduced the protein level of Beclin-1 (Fig. 4C and D). ULK1 and p-ULK1 protein levels were not significantly different between Huh7-R cells transfected with siHOTAIRM1 and those transfected with the NC sequence1 (Fig. 4C and D, Additional file 4: Fig. S4B).

### Expression of HOTAIRM1 and key autophagy-related molecules in HCC patients with different lenvatinib sensitivities

To verify the relationship between HOTAIRM1 and key autophagy-related molecules in the real world, the expression levels of HOTAIRM1, Beclin-1 and p62 in different lenvatinib-sensitive HCC patients were detected by RT-qPCR. The results showed that HOTAIRM1 was significantly more highly expressed in lenvatinib-resistant HCC patients than in lenvatinib-sensitive HCC patients (Fig. 4E). Under the same standards and conditions, the expression levels of the key autophagy-related molecules Beclin-1 and p62 were also detected. The level of Beclin-1 in lenvatinib-resistant HCC patients was significantly higher than that in lenvatinib-sensitive patients (Fig. 4F), whereas the trends of p62 level was reverse (Fig. 4G).

### MiR-34a may be a crucial mediator in the HOTAIRM1-Beclin-1 signaling pathway

**Fig. 5:**
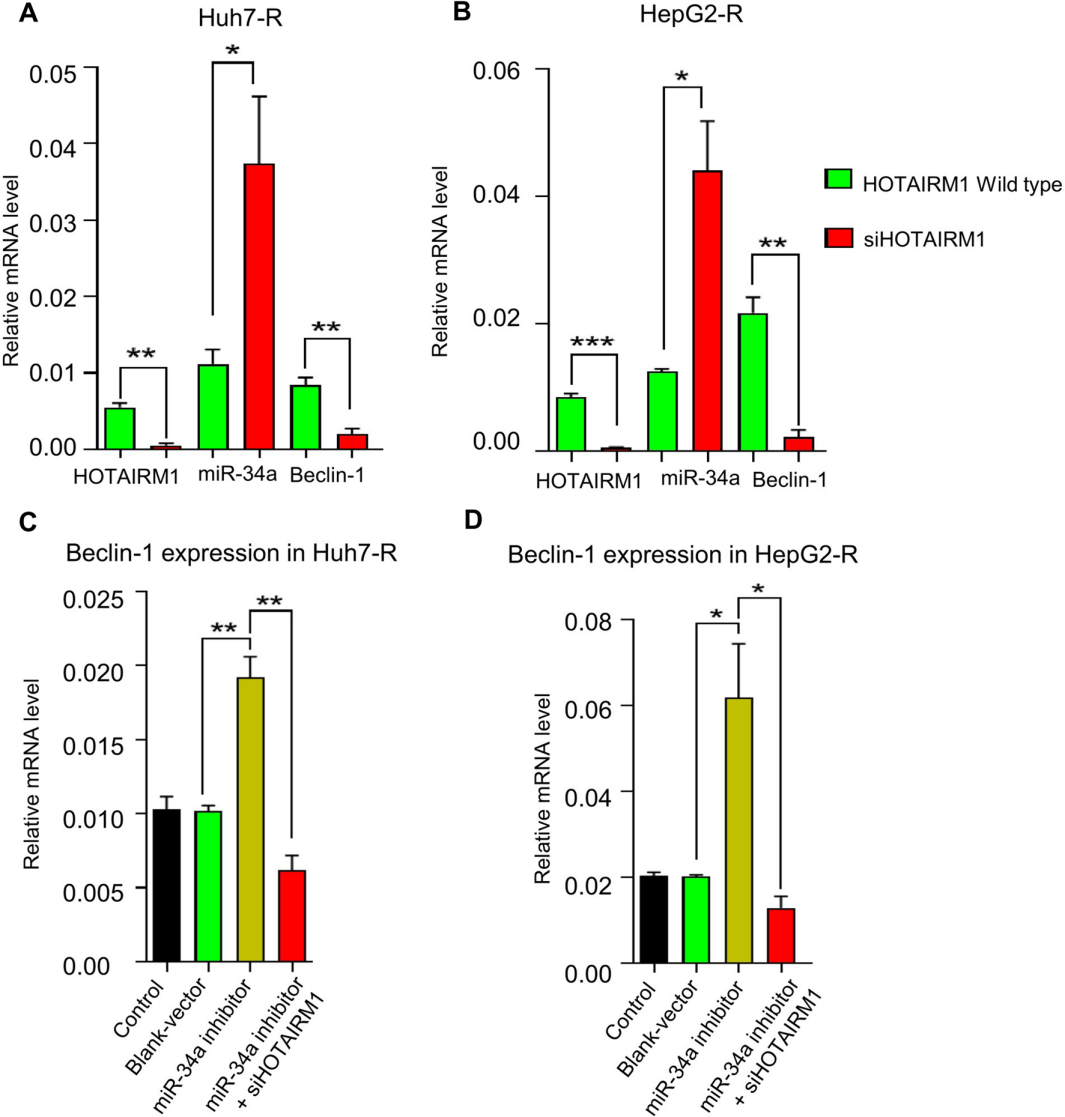
Resistance of HCC to lenvatinib is regulated by the lncRNA HOTAIRM1-miR-34a-beclin-1 axis. Knockdown of HOTAIRM1 increased miR-34a expression and reduced beclin-1 expression in lenvatinib-resistant Huh7-R (**A**) and HepG2-R (**B**) cells. Downregulation of miR-34a increased beclin-1 expression, and the phenotype was reversed by knocking down of HOTAIRM1 in lenvatinib-resistant Huh7-R (**C**) and HepG2-R (**D**) cells. * P < 0.05, ** P < 0.01, *** P < 0.001.

To predict the key molecules that mediate the effect of lncRNAs on autophagy, the bioinformatics methods of GO and pathway analyses were used to analyze the relationship between HOTAIRM1 and the autophagy-associated gene Beclin-1. RT-qPCR was used to detect the expression of miR-34a. It was demonstrated that knocking down HOTAIRM1 in lenvatinib-resistant cell lines increased the level of miR-34a and inhibited the expression of Beclin-1 in Huh7-R (Fig. 5A) and HepG2-R cells (Fig. 5B). To further verify the relationship between miR-34a and Beclin-1 in lenvatinib-resistant HCC, miR-34a inhibitors were used to downregulate miR-34a, and the NC sequence was used as a control. The results showed that knockdown of miR-34a significantly increased the expression level of Beclin-1 in both Huh7-R (Fig. 5C) and HepG2-R cells (Fig. 5D). However, this phenotype was easily rescued by knocking down HOTAIRM1 (Fig. 5C and D). These data indicated that the resistance of HCC to lenvatinib is contributed mainly by the HOTAIRM1-miR-34a-beclin-1 axis. Importantly, miR-34a has been reported to target the 3'-UTR of the autophagy activation-related genes HMGB1 and ATG9A [22]. Therefore, further study is urgently needed to validate how miR-34a negatively regulates Beclin-1 and ultimately affects the HOTAIRM1-Beclin-1 pathway.

### Downregulation of HOTAIRM1 combined with an autophagy inhibitor enhanced lenvatinib sensitivity in drug-resistant HCC

**Fig. 6:**
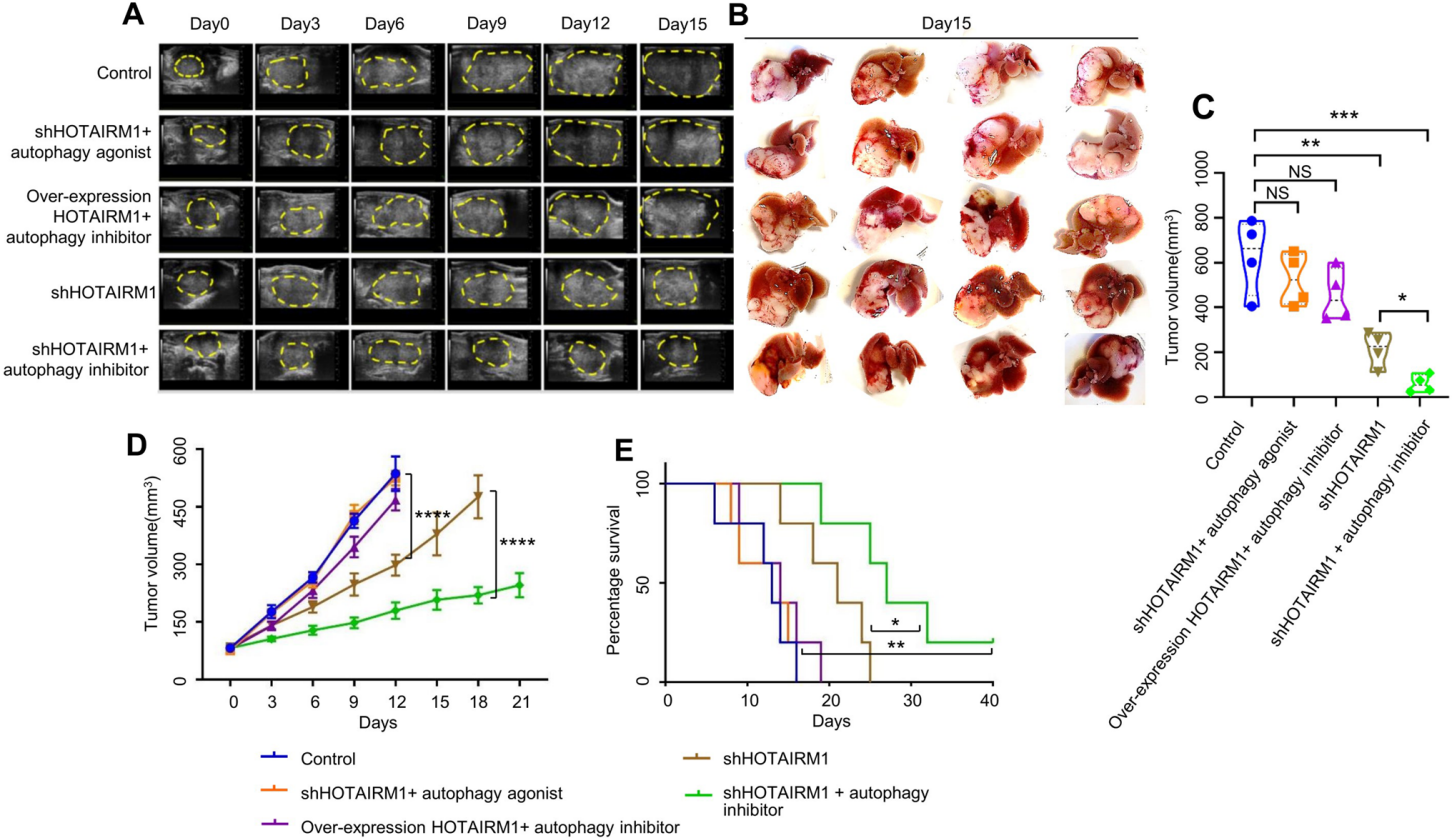
Knockdown of HOTAIRM1 combined with an autophagy inhibitor reversed lenvatinib-resistant HCC to lenvatinib-sensitive HCC in vivo. High-frequency ultrasound images of the Huh7-R orthotopic tumor-bearing nude mice on days 0, 3, 6, 9, 12 and 15 (**A**, N = 5). Naked-eye observation of images of the Huh7-R orthotopic tumor-bearing nude mice on day 15 (**B**, N = 4). Tumor growth profile of each indicated treatment group on day 15 (**C**, N = 4). Tumor growth profile of each indicated treatment group on days 0, 3, 6, 9, 12, 15, 18 and 21 (**D**, N = 5). Survival data from the Huh7-R orthotopic mouse model (**E**, N = 5). NS, no significant difference; *P < 0.05; **P < 0.01; ***P < 0.001; ****P < 0.0001

To determine whether the HOTAIRM1 effect on lenvatinib sensitivity was dependent on the activation of autophagy, we next evaluated the antitumor efficacy by combining the downregulation of HOTAIRM1 and an autophagy inhibitor in an orthotopic xenograft nude mouse model. The mice in the autophagy inhibitor group were intraperitoneally injected with 3-MA (20 mg/kg/day, dissolved in DMSO, 5 mice/group) or vehicle. The mice in the autophagy agonist group were given intraperitoneal injections of 1.5 mg/kg rapamycin or vehicle every 3 days (5 mice/group). The mice in the overexpression HOTAIRM1 group were seeded with overexpression HOTAIRM1 Huh7-R cells (5 mice/group). The mice in the downregulated HOTAIRM1 group were seeded with shHOTAIRM1 Huh7-R cells (5 mice/group). Tumor-bearing mice were treated with lenvatinib alone (as the control group) or with lenvatinib combined with an autophagy agonist or inhibitor. Tumor growth was monitored by high-frequency ultrasound imaging (Fig. 6A). In vivo results revealed that autophagy agonist treatment with shHOTAIRM1 or autophagy inhibitor therapy with overexpression of HOTAIRM1 did not inhibit HCC growth in those mice compared to control mice, but the combination of autophagy inhibitor and shHOTAIRM1 or even shHOTAIRM1 alone was significantly more effective than other treatments (naked-eye observation of images of the individual Huh7-R orthotopic tumor on day 15 in Fig. 6B, mean tumor volumes on day 15 in Fig. 6C, and mean tumor volumes on days 0, 3, 6, 9, 12, 15, 18, 21 in Fig. 6D). Notably, the group treated with autophagy inhibitor combined with shHOTAIRM1 or even shHOTAIRM1 alone showed a significant and substantial survival benefit (median overall survival of 28 days and 20 days, respectively, almost double that in the other groups in this model, p = 0.0018 and p = 0.0088) (Fig. 6E). Moreover, the group treated with autophagy inhibitor combined with shHOTAIRM1 had a better outcome than the group treated with shHOTAIRM1 alone, which also showed a significant and substantial survival benefit (p = 0.0357) (Fig. 6E).

It can be concluded that knockdown of HOTAIRM1 can significantly increase the sensitivity of lenvatinib-resistant strains to lenvatinib, but this effect of lenvatinib sensitivity is reduced when an autophagy agonist is added. Conversely, overexpression of HOTAIRM1 significantly reduced the sensitivity of the parental cells to lenvatinib but increased the lenvatinib sensitivity after adding an autophagy inhibitor. These findings suggest that downregulation of HOTAIRM1 combined with an autophagy inhibitor can markedly improve the efficacy of lenvatinib therapy in lenvatinib-resistant HCC.

## Discussion

LncRNAs are RNA molecules involved in gene regulation at the transcriptional, posttranscriptional, and epigenetic levels. LncRNAs also play an important role in many pathophysiological processes of cancer and may be novel therapeutic targets in cancer therapy [23–25]. However, the mechanism by which lncRNAs affect drug resistance is currently unclear [11]. Some lncRNAs affect the cancer cell cycle by regulating apoptosis while others mediate abnormal metabolism in cancer cells and make cancer cells off-target [26, 27]. The latest study reported that lncRNAs participate in the process of epithelial-mesenchymal transition of cancer cells and affect cancer cell drug resistance [28]. In addition, lncRNA-MEG3, a tumor suppressor gene, regulates the occurrence and progression of bladder cancer by inhibiting autophagy [29]. The latest study has shown that overexpression of HULC increases the expression of LC3-II, that is, activates autophagy in SGC7901 cells [30].

LncRNA HOTAIRM1 is a highly conserved HOX gene cluster that may promote tumorigenesis in adult tissue cells by regulating many biological functions, such as apoptosis and cell differentiation [31, 32]. HOTAIR has been associated with proliferation and invasion in endometrial cancer [33]. Increased expression of HOTAIR in HCC is thought involved in metastatic progression by several pathways correlated to cell adhesion [34]. Zhou et al. demonstrated that HOTAIR may have a critical role in the proliferation of HCC by regulating cell cycle, cyclin D1 expression and STAT3 activity [35]. Besides its important role in tumorigenesis, the latest report suggest that the high expression level of HOTAIR in HCC is associated with disease progression, could be a candidate biomarker for predicting tumor recurrence in HCC patients and might be a potential therapeutic target [36–39]. However, there are no report on the relevant roles of HOTAIRM1 in HCC and lenvatinib resistance.

In this study, our results showed that HOTAIRM1 is the most significantly highly expressed lncRNA in lenvatinib-resistant cells. Knocking down of HOTAIRM1 can reverse lenvatinib-resistant HCC to lenvatinib-sensitive HCC. In recent years, researchers have verified that the autophagy signaling pathway impacts the efficiency of chemotherapy and the prognosis of patients with cancer. Some studies have also shown that lncRNAs play an important role in tumor autophagy. Chen et al. demonstrated that HOTAIRM1 upregulates autophagy by regulating related miRNAs targeting ULK1 [40]. HOTAIR was also reported significantly overexpressed in HCC and activate autophagy by upregulating ATG7 and ATG3, promoting Promote disease progression [41]. In addition, HOTAIR can regulate the cisplatin-sensitivity of endometrial cancer via the regulation of autophagy by affecting Beclin-1 expression [42]. In this study, in vitro experiments demonstrated that knocking down HOTAIRM1 significantly reduced the expression of LC3II/I while increasing the protein level of p62 in lenvatinib-resistant HCC. Further experiments showed that Beclin-1 was highly expressed in lenvatinib-resistant HCC and positively regulated by HOTAIRM1. The relationship between HOTAIRM1 and the key autophagy-related molecule Beclin-1 was also validated in real-world HCC patients. Notably, these data further suggest that HOTAIRM1 may trigger the autophagy pathway and lead to lenvatinib resistance in HCC.

MicroRNAs (miRNAs) are small endogenous non-coding RNAs which was thought to affect drug resistance. HOTAIR was proved to induce sorafenib resistance through suppressing miR-217 in HCC [43]. While MiR-34a is another miRNA which was demonstrated to target several molecules associated with the cell proliferation, cell cycle and cell survival. Dysregulation of MiR-34a will affect drug sensitivity in plenty cancers. Duan et al. reported that LncRNA HOTAIR induce HCC cells resistance to Taxol via activating AKT phosphorylation by down-regulating miR-34a [44]. Of note, Yang et al. reported that MicroRNA-34a can regulate Bcl-2 expression and sensitizes HCC to sorafenib [45]. However, the correlation between miR-34a expression and lenvatinib resistance has not been explored in HCC yet.

**Fig. 7:**
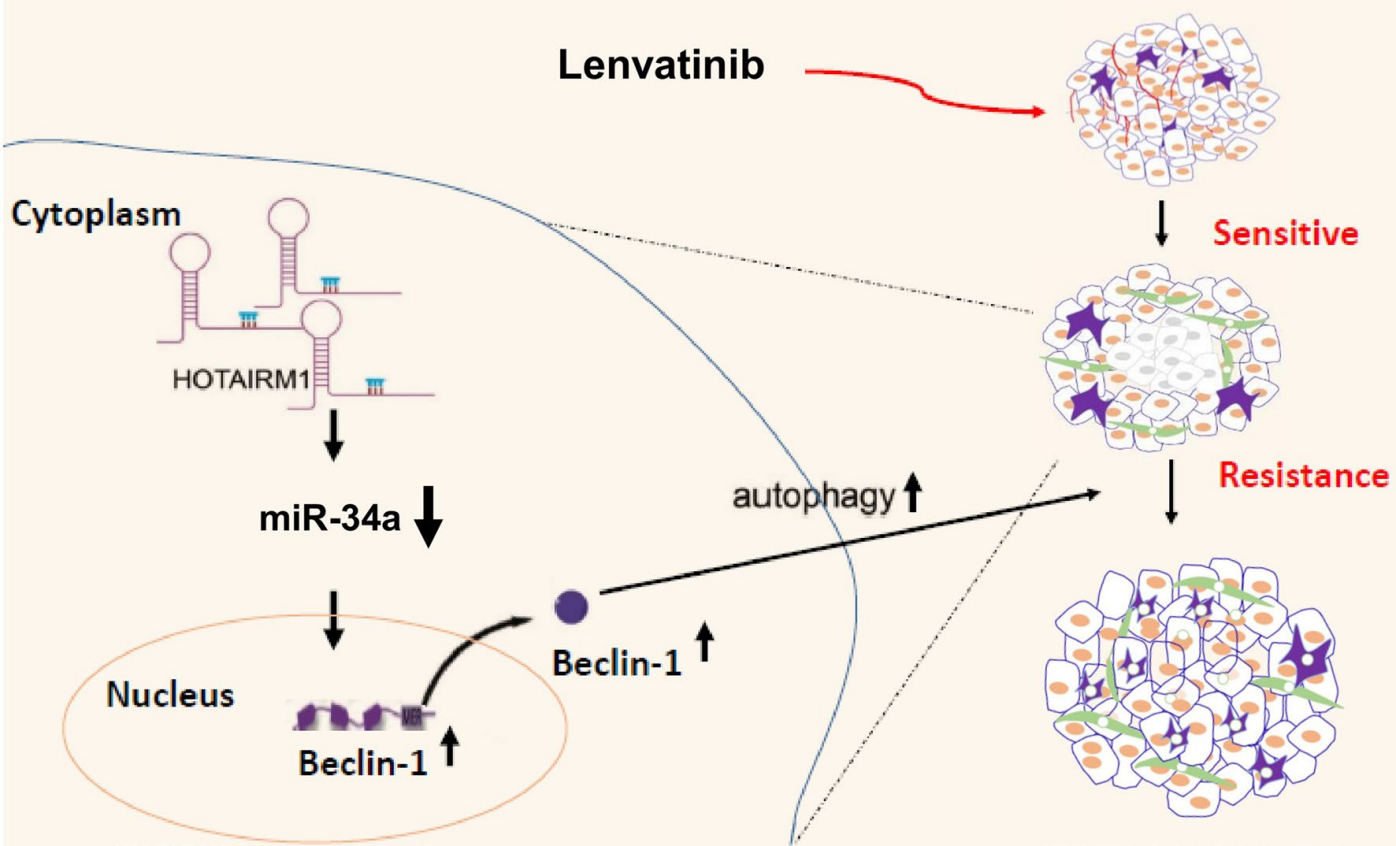
Schematic indicating roles of HOTAIRM1 and autophagy signaling in HCC cells experiencing lenvatinib resistance. Overexpression of the lncRNA HOTAIRM1 promotes lenvatinib resistance by downregulating miR-34a and activating autophagy in HCC

In our current study, knockdown of miR-34a significantly increased the expression level of Beclin-1 in lenvatinib-resistant HCC. However, this phenotype can easily be rescued by knocking down HOTAIRM1. These data indicated that the resistance of HCC to lenvatinib is contributed mainly by the HOTAIRM1-Beclin-1 axis via downregulation of miR-34a (Fig. 7).

To further validate the HOTAIRM1 effect on lenvatinib sensitivity was dependent on the activation of autophagy, our in vivo study demonstrated that downregulation of HOTAIRM1 combined with an autophagy inhibitor can markedly improve the efficacy of lenvatinib therapy in lenvatinib-resistant HCC. However, it is rather frustrated that in this orthotopic transplantation tumor model, combined treatment with knockdown of HOTAIRM1 plus autophagy inhibitor and lenvatinib could inhibiting tumor growth and prolong survival of animals for a short period of time. The reason may be closely related to the complex tumor microenvironment and signal pathways. Of note, HOTAIRM1 was also reported involved in acquired chemotherapy resistance. Knocking down of HOTAIRM1will down-regulated HOXA1 expression and restored sensitivity to tamoxifen in tamoxifen-resistant MCF7 (TAMR) cells [17]. While in other studies, Ren et al.demonstrated that HOTAIRM1 might act as a tumor-suppressor in 5-FU resistant colorectal cancer cells and inhibit multi-drug resistance [18].

However, our study has some limitations. Firstly, the clinical data used in this study were acquired from a relatively small cohort, so selection bias or potential biases related to imbalanced clinical characteristics is inevitable. Secondly, when combined treatment with knockdown of HOTAIRM1 could inhibiting tumor growth and prolong survival of animals for only a short period of time. More prospective works and experimental research should be conducted for optimizing and evaluating a combination in the study of HOTAIRM1. Thirdly, how HOTAIRM1 downregulate miR-34a and activate autophagy is indeterminacy. Further research on the regulation of autophagy by the HOTAIRM1-related signaling pathway is need and will help to elucidate the new mechanism of lenvatinib resistance, providing a theoretical basis for the prediction of lenvatinib therapy sensitivity in HCC and the development and application of new therapeutic targets for reversing lenvatinib resistance.

## Conclusion

The current research demonstrated that HOTAIRM1 is an independent drug resistance factor, significantly associated with the efficacy of lenvatinib in HCC and helpful for guiding HCC patient treatment decision-making as well as follow-up visits. Further studies revealed a new role for HOTAIRM1 in the critical downregulation of miR-34a and upregulation of Beclin-1, leading to activation of autophagy, thereby inducing lenvatinib resistance in HCC. These findings are therefore proposed to help identify new targets for HCCs novel combination therapeutics to overcome lenvatinib resistance (Additional files 5, 6).

## Supplementary Information

**Additional file 1. **Proliferation inhibitory effect of lenvatinib on lenvatinib-resistant (HepG2-R and Huh7-R) and parental HCC cells (HepG2 and Huh7). Lenvatinib (1 μmol/L) significantly inhibited the proliferation of Huh-7 cells on day 7(A), and 2 μmol/L lenvatinib significantly inhibited the proliferation of HepG2 cells on day 7(B). Lenvatinib at the same concentration could not inhibit the proliferation of lenvatinib-resistant Huh7-R and HepG2-R cells.

**Additional file 2. **Differential expression levels of lncRNAs in lenvatinib-resistant (HepG2-R and Huh7-R) and parental HCC cells (HepG2 and Huh7) by RT‒qPCR. LncRNA HOTAIRM1 was significantly highly expressed in lenvatinib-resistant Huh7-R (A) and HepG2-R (B) cells. * P < 0.05 and *** P < 0.001, NS, no significant difference.

**Additional file 3. **Upregulate of HOTAIRM1 can reverses lenvatinib-sensitive HCC to lenvatinib-resistance HCC. RT‒qPCR results showed that the expression level of HOTAIRM1 was significantly upregulated after transfection of HOTAIRM1 overexpression lentiviruses to lenvatinib-sensitive cells compared with the control cells (A and C). MTT assay demonstrated the inhibitory effect of lenvatinib on cell proliferation with and without HOTAIRM1 overexpression lentiviruses transfection. And this effect can be reversed when autophagic flux inhibitors CQ was added (B and D). *** P < 0.001, NS, no significant difference.

**Additional file 4. **Western blot results of p-ULK1/ULK1. The levels of p-ULK1/ULK1 in HCC with different lenvatinib sensitivities (A). The levels of p-ULK1/ULK1 between Huh7-R cells transfected with siHOTAIRM1 and those transfected with the NC sequence1 (B).

**Additional file 5. **Knockdown of HOTAIRM1 combined with an autophagy inhibitor reversed lenvatinib-resistant HCC to lenvatinib-sensitive HCC in vivo. Tumor volume of each indicated treatment mice on days 0, 3, 6, 9, 12, 15, 18 and 21 (N = 5).

**Additional file 6. **Knockdown of HOTAIRM1 combined with an autophagy inhibitor reversed lenvatinib-resistant HCC to lenvatinib-sensitive HCC in vivo. Tumor volume of each indicated treatment mice on day 15 (N = 4).

## Data Availability

The datasets used and analyzed during the current study are available from the corresponding author on reasonable request.
