# Supplementary figures and images for: Overexpression of the lncRNA HOTAIRM1 promotes lenvatinib resistance by downregulating miR-34a and activating autophagy in hepatocellular carcinoma
Source: Discov Oncol. 2023 May 12;14:66. doi: 10.1007/s12672-023-00673-8 (PMC10182232; doi:10.1007/s12672-023-00673-8)

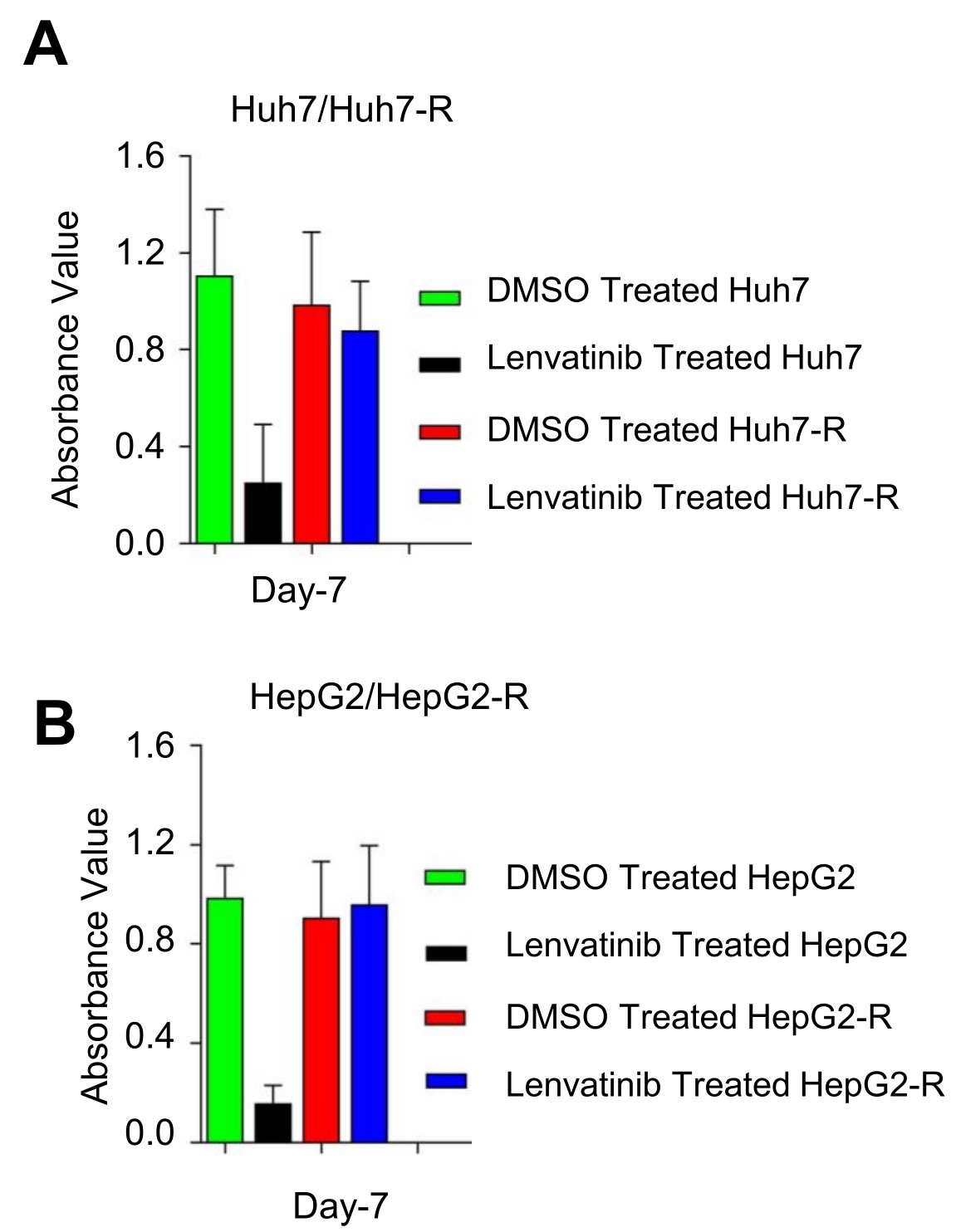

Supplement: Supplementary file 1 — Additional file 1. Proliferation inhibitory effect of lenvatinib on lenvatinib-resistant (HepG2-R and Huh7-R) and parental HCC cells (HepG2 and Huh7). Lenvatinib (1 μmol/L) significantly inhibited the proliferation of Huh-7 cells on day 7(A), and 2 μmol/L lenvatinib significantly inhibited the proliferation of HepG2 cells on day 7(B). Lenvatinib at the same concentration could not inhibit the proliferation of lenvatinib-resistant Huh7-R and HepG2-R cells. [file 12672_2023_673_MOESM1_ESM.jpg]

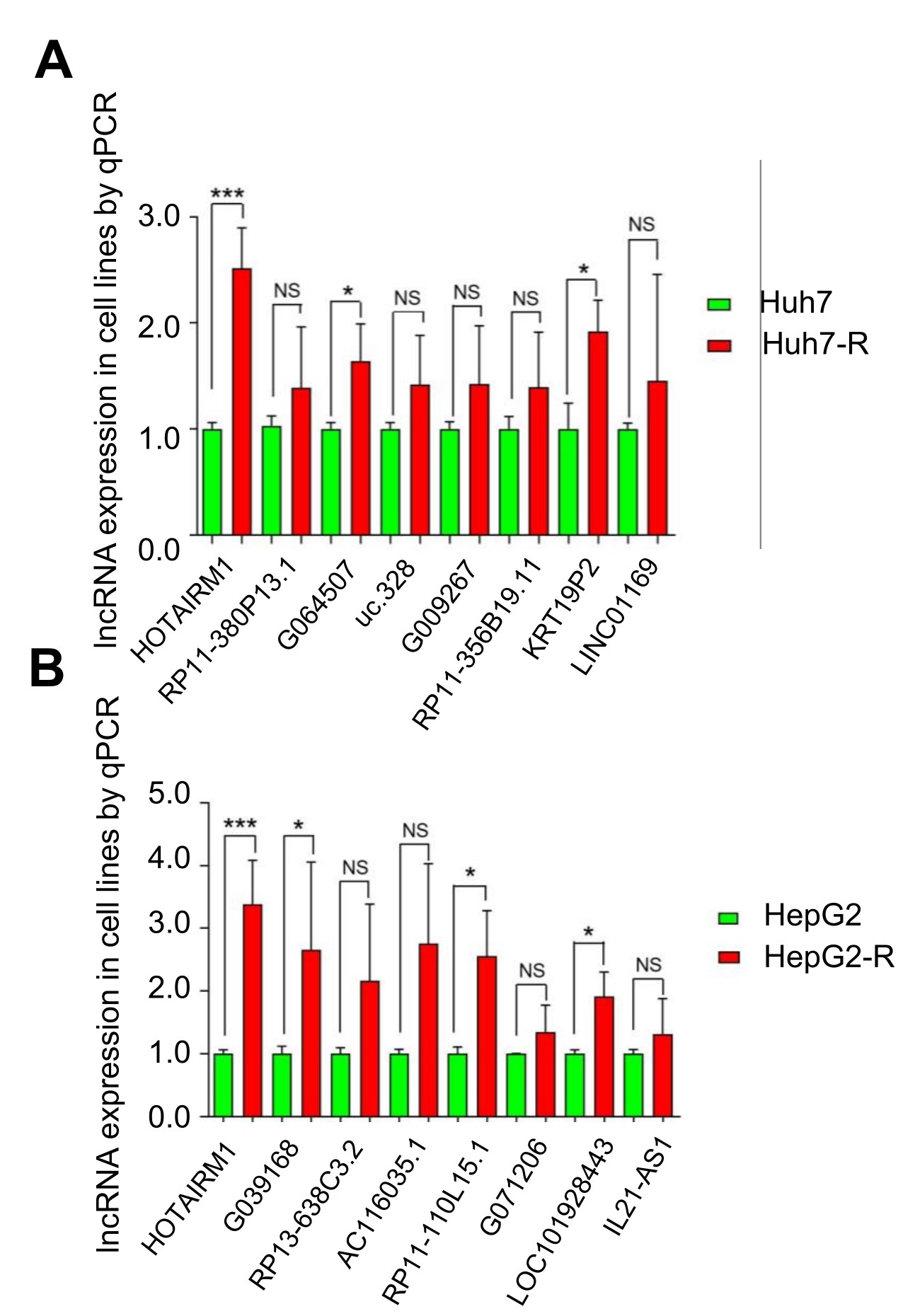

Supplement: Supplementary file 2 — Additional file 2. Differential expression levels of lncRNAs in lenvatinib-resistant (HepG2-R and Huh7-R) and parental HCC cells (HepG2 and Huh7) by RT‒qPCR. LncRNA HOTAIRM1 was significantly highly expressed in lenvatinib-resistant Huh7-R (A) and HepG2-R (B) cells. * P < 0.05 and *** P < 0.001, NS, no significant difference. [file 12672_2023_673_MOESM2_ESM.jpg]

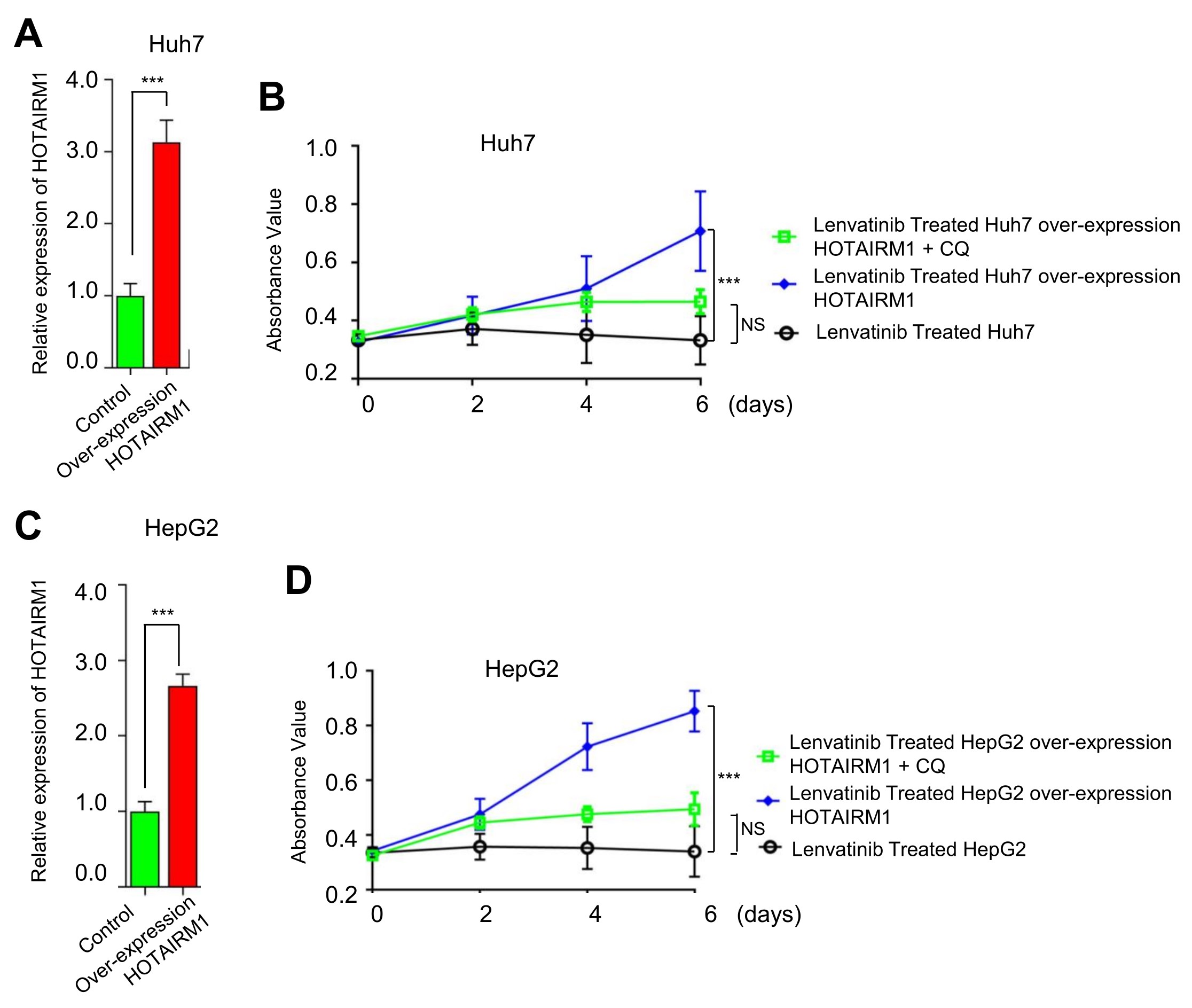

Supplement: Supplementary file 3 — Additional file 3. Upregulate of HOTAIRM1 can reverses lenvatinib-sensitive HCC to lenvatinib-resistance HCC. RT‒qPCR results showed that the expression level of HOTAIRM1 was significantly upregulated after transfection of HOTAIRM1 overexpression lentiviruses to lenvatinib-sensitive cells compared with the control cells (A and C). MTT assay demonstrated the inhibitory effect of lenvatinib on cell proliferation with and without HOTAIRM1 overexpression lentiviruses transfection. And this effect can be reversed when autophagic flux inhibitors CQ was added (B and D). *** P < 0.001, NS, no significant difference. [file 12672_2023_673_MOESM3_ESM.jpg]

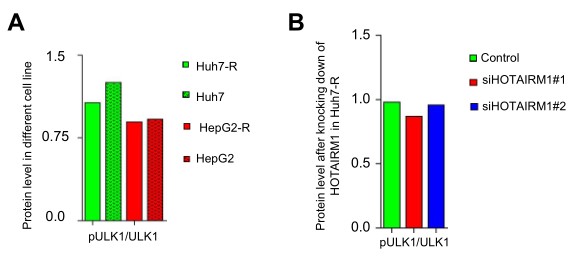

Supplement: Supplementary file 4 — Additional file 4. Western blot results of p-ULK1/ULK1. The levels of p-ULK1/ULK1 in HCC with different lenvatinib sensitivities (A). The levels of p-ULK1/ULK1 between Huh7-R cells transfected with siHOTAIRM1 and those transfected with the NC sequence1 (B). [file 12672_2023_673_MOESM4_ESM.jpg]
